# Prevalence of hepatitis B surface antigen (HBsAg) in a blood donor population born prior to and after implementation of universal HBV vaccination in Shenzhen, China

**DOI:** 10.1186/s12879-016-1834-2

**Published:** 2016-09-20

**Authors:** Zhen Wang, Jinfeng Zeng, Tingting Li, Xin Zheng, Xiaoxuan Xu, Xianlin Ye, Liang Lu, Weigang Zhu, Baocheng Yang, Jean-Pierre Allain, Chengyao Li

**Affiliations:** 1 Department of Transfusion Medicine, Southern Medical University, Guangzhou, 510515 China; 2 Shenzhen Blood Center, Shenzhen, China; 3 Baoan Central Blood Station, Shenzhen, China; 4 Department of Haematology, University of Cambridge, Cambridge, UK; 5 School of Public Health and Tropical Medicine, Southern Medical University, Guangzhou, China

**Keywords:** HBV, Prevalence, Vaccination, Blood donors, China

## Abstract

**Background:**

Neonatal hepatitis B vaccination program at birth has been implemented nationwide since 1992 in China. However, current HBV prevalence status in blood donors has not been entirely examined, which may impact HBV safety in blood donations as the vaccinees over 18 years old progressively become the majority population of blood donors.

**Methods:**

In this study, 569,145 blood donors were screened for HBsAg by rapid tests and enzyme immunoassays, among them 475,538 blood samples with negative HBsAg were further screened for HBV DNA by nucleic acid testing between 2005 and 2014 at Shenzhen blood center.

**Results:**

An overall 2.3 % HBsAg prevalence was found in the blood donor population during the past 10 years (2.86 % in 2005, 1.76 % in 2010, and 2.79 % in 2014, respectively). HBsAg seroconversion occurred in 0.37 % of repeat-donors. When stratified by age, the prevalence of HBsAg was found significantly higher in younger donors age 18–25 years (2.73 %) than in those 26–35 years (2.13 %), 36–45 years (2.03 %) and 46–58 years (1.71 %) (*P* < 0.001), unexpectedly suggesting that younger donors remained at risk of chronic HBV infection. Assuming that donors aged 18–22 born before or after 1992 were non-vaccinated and vaccinated, respectively, HBsAg prevalence was higher in first-time donors born ≥1992 (3.9 %) than prior to 1992 (3.5 %, *P* = 0.005). The incidence of HBV infection in the 5-year period examined was significantly lower in repeat-donors born ≥1992 (0.27 %) than prior to 1992 (0.6 %, *P* = 0.008). The yield of HBV DNA+/HBsAg- donors was 1:3,302, including 1:4,486 occult infections and 1:43,231 window period infections.

**Conclusion:**

Young blood donors born after implementation of universal HBV vaccination in China presented higher prevalence of HBsAg but lower incidence of HBsAg seroconversion than older, presumed unvaccinated, donors. HBV vaccine boosting for adolescents at 15–17 years old prior to reaching blood donor age might help improve blood safety.

## Background

Hepatitis B virus (HBV) infection is a global threat to human health, especially in developing countries with high prevalence. Individuals with detectable surface antigen at six months interval (HBsAg) are considered carriers of HBV chronic infection. In China, the prevalence of chronic HBV carriers declined from 9.5 to 7.2 % in the general population aged 1–59 years. In children <5 years between 1992 and 2006 HBsAg prevalence was 1 % following nationwide implementation of universal HBV vaccination at birth [1]. However, although HBV vaccine compliance progressively increased between 1992 and 2000 from 30 to 76 % then stabilized up to 96 % in 2005, recent reports indicated an overall compliance of >90 % in general populations <20 years of age corresponding to a significant decline in HBsAg prevalence between 3.6 % below 20 and 10.2 % between 20 and 60 years of age [2, 3]. Screening of HBsAg in blood donors massively decreased the risk of HBV transmission by transfusion, but did not identify pre-seroconversion window period infection (WPI) [4]. In recent years, occult HBV infection (OBI) was defined as an absence of detectable HBsAg in circulation but presence of HBV DNA in blood or liver tissue [5]. Implementation of nucleic acid testing (NAT) for HBV DNA detection yielded 1:1000-1:20,000 donor OBI carriers [4]. However, a small number of blood donors with low-level of HBV DNA load could not be identified by current screening NAT due to insufficient sensitivity [6, 7].

Over the past decades, universal vaccination has led to a 70–90 % decrease in chronic HBV carrier rates worldwide [8], and has resulted in a substantial decline in disease burden, hepatitis B-related morbidity and mortality [9–11]. Since infant vaccination started nationwide in 1992, HBV vaccinees are gradually becoming the majority of blood donors in China and are expected to significantly improve HBV safety in blood donations. However, in a previous study, we reported cases of HBV recovered, chronic and occult infections in vaccinated blood donors [12]. The current trend of HBV infection in the blood donor population has not been systematically analyzed. In this study, we conducted a comprehensive survey and analysis of HBsAg screening results during the past 10 years (2005 to 2014) in donors stratified according to type, geographical origin, gender, age, and assumed vaccination status.

## Methods

### Subjects and samples

Candidate blood donors who were recruited for giving blood in Shenzhen between 2005 and 2014 were enrolled in this study. All donors were voluntary and non-remunerated. First-time blood donors were defined as donors who gave blood for the first time, while repeat-donors were defined as donors who donated blood more than once at the Shenzhen blood center. The donors born in Guangdong province were classified as native donors, while the donors born in other provinces were classified as donors of other origins. Blood samples (sera or plasmas) were collected from blood donors at the Shenzhen blood center or during mobile collection within 20 km of the city center.

### Serological testing

All candidate donors passed the pre-donation questionnaire and underwent rapid pre-donation testing for HBsAg (dipsticks with colloidal gold strip method, Abon Diagnostics, Hangzhou, China) and ALT (Roche Refletron, Roche Diagnostics Gmbh, Mannhein, Germany) at the blood collection sites. HBsAg reactive samples were considered confirmed. After pre-donation testing, blood was collected from the qualified donors, and samples were further screened in parallel and duplicate with two different HBsAg EIAs (Diasorin S.P.A.-UK Branch and Xinchang Diagnostics, Xiamen, China), anti-HCV (Ortho Clinical Diagnostics, UK and Lizhu Diagnostics, Zhuhai, China), anti-HIV1/2 (Biorad, Marnes-la-Coquette, France and Wantai Diagnostics, Beijing, China), Syphilis (Diasorin S.P.A.-UK Branch and Lizhu Diagnostics, Zhuhai, China), respectively [12–14]. Alanine aminotransferase (ALT) level was quantified with a kinetic method (AusBio Biotech., China). Samples HBsAg reactive with both EIAs were considered confirmed, while samples HBsAg reactive with a single EIA were re-tested twice by the reactive assay in duplicate. If the testing was repeatable, the sample was considered HBsAg positive. Donor samples non-reactive with serological tests and with normal ALT level were further screened with the Triplex NAT for HIV-1, HCV and HBV genomes as previously described [13].

### Nucleic acid testing

Detection of viral nucleic acids with NAT was performed using three different modalities: Roche Cobas Ampliscreen assay (MP24-NAT, Roche Molecular Diagnostics, Raritan, USA) was used between January 2005 and January 2006, Kehua real-time PCR assay (MP8-NAT, Kehua Biotechnology Ldt., Shanghai, China) between February 2006 and January 2009 and Procleix Ultrio assay (ID-NAT, Novartis Diagnostics, Emeryville, USA) between February 2009 and December 2014, respectively according to manufacturers’ recommendations. Between January 2005 and January 2009 NAT was applied to mini-pool of 24 or 8 samples (MP24 or MP8-NAT) and since February 2009 to individual samples (ID-NAT). Nucleic acids were extracted from 500 μl of individual plasma samples. Reactive samples were further qualified with the discriminatory assays (COBAS Monitor Test, in-house PCR or discriminatory Procleix Ultrio test) for the virus responsible for the signal (HBV, HCV or HIV-1).

### Follow-up testing

Blood donors who tested HBV DNA positive but HBsAg negative (HBV DNA+/ HBsAg-) were further tested in 1–3 follow-up samples for HBV DNA and serological markers. Anti-HBs, HBeAg, anti-HBe and anti-HBc were tested with HBV EIAs (Kehua, Shanghai, China). Anti-HBs was quantified in IU/L with anti-HBs electrochemiluminescence immunoassay (Roche), while HBV DNA load was quantified in IU/ml by in-house real-time PCR as previously described [12, 14].

### Statistical analyses

SPSS software (version 16.0) was used. Categorical variables were compared by using Fisher’s exact test. *P* < 0.05 were considered statistically significant.

## Results

### Demographic characteristics of blood donors

**Fig. 1:**
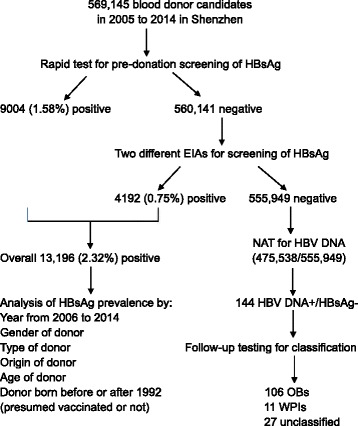
Flow chart of blood donor testing. The first part of the study consisted in retrieving HBsAg screening results for both candidate donors and selected donors. The second part was the detection of HBV DNA in HBsAg negative donors

**Fig. 2:**
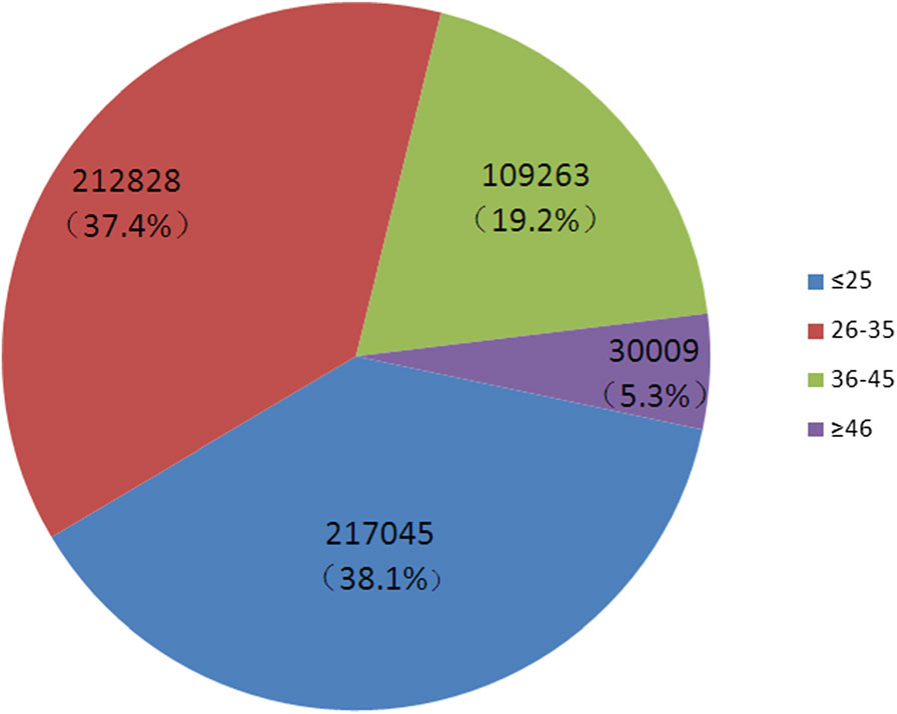
Distribution of blood donors according to ages. Percentages (%) of 568,114 blood donors from 2005 to 2014 were distributed according to population age groups

Between 2005 and 2014, a total of 569,145 candidate blood donors were recruited and selected by Shenzhen blood center, Shenzhen, Guangdong, China, from whom 724,700 blood donations were collected from pre-donation testing qualified donors (Table 1, Fig. 1). The number of blood donations almost doubled between 2005 and 2014. Of those blood donors, 66.7 % were male and 33.3 % female, 60.5 % were first-time donors and 39.5 % repeat-donors, respectively. The ratio between native Guangdongese to donors of other origins (non-Guangdongese) was 1:2.3, indicating that migrants from other provinces contributed 70 % of blood donations. Donor age ranged between 18 and 58 years stratified into 4 age groups of 18–25, 26–35, 36–45 and 46–58 years (Fig. 2). Seventy-six % of blood donations were collected from young donors 18–25 or 26–35 years.

**Table 1:** Demographic characteristics of candidate blood donors between 2005 and 2014 in Shenzhen, China

Year	Number of donors	% of donors in various categories						Number of donations
		First-time	Repeat	Male	Female	Native^a^	Other origins^a^	
2005	42,638	67.5	32.5	68.1	31.9	27.7	72.3	52,398
2006	44,411	64.6	35.4	69.1	30.9	28.8	71.2	55,117
2007	45,248	50.2	49.8	68.2	31.8	27.4	72.6	58,051
2008	47,086	60.0	40.0	68.5	31.5	26.7	73.3	60,794
2009	48,341	58.8	41.2	68.3	31.7	27.9	72.1	64,496
2010	57,248	62.1	37.9	66.2	33.8	28.4	71.6	73,953
2011	66,232	61.4	38.6	66.2	33.8	30.4	69.6	83,353
2012	73,194	63.8	36.2	66.3	33.7	31.6	68.4	92,033
2013	72,314	56.2	43.8	66.1	33.9	32.2	67.8	92,125
2014	72,433	60.6	39.4	63.4	36.6	33.8	66.2	92,380
Total	569,145	60.5	39.5	66.7	33.3	29.9	70.1	724,700

^a^According to the initial identity card registration that indicates the birth place, blood donors were classified as native born in Guangdong province or of other origins (born in other provinces of China)

### Prevalence of HBV in blood donor population

**Fig. 3:**
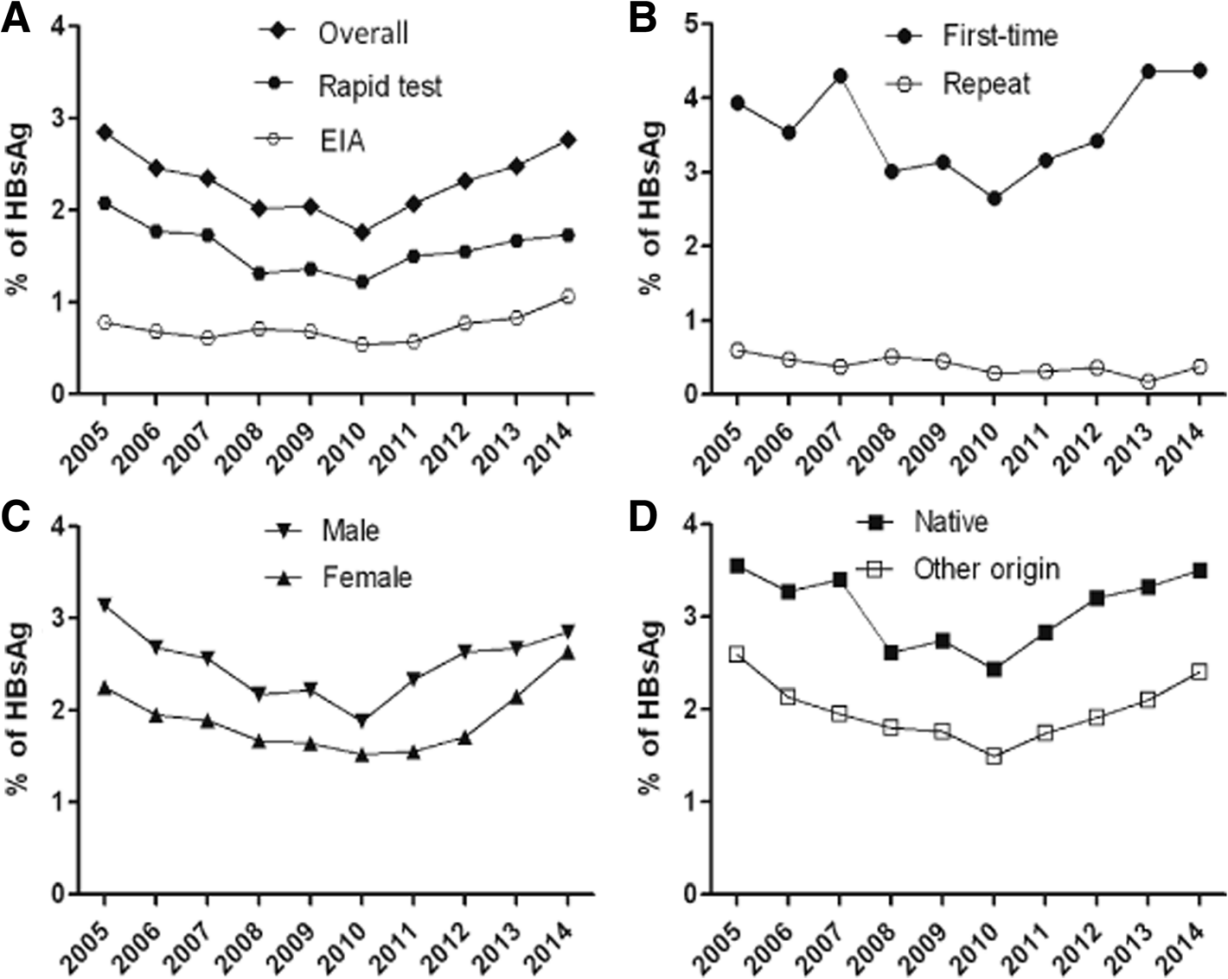
Prevalent trends of HBsAg in blood donor populations. **a** Overall prevalence of HBsAg detected by rapid tests (dipsticks) and EIAs. **b** HBsAg prevalence in first-time and repeat donors. **c** HBsAg prevalence in male and female donors. **d** HBsAg prevalence in native Guangdongese and other non-Guangdongese donors. *P* values were calculated as <0.0001 between the first-time and repeat, male and female, native and other original donors, respectively

Among 569,145 candidate blood donors, 9004 (1.58 %) were HBsAg positive in pre-donation screening by the dipstick rapid test, and 4192 (0.75 %) of 560,141 selected donors were further identified as HBsAg reactive by EIA after donation. Overall, HBsAg prevalence was 2.32 % in blood donors over the past 10 years. Year by year HBsAg prevalence in candidate donors is presented in Fig. 3a. It follows a U-type curve with, 2.86 % prevalence in 2005 declining to 1.76 % in 2010, and regularly increasing up to 2.77 % in 2014. When data is stratified according to donor type (first-time or repeat), gender or geographical origin (birth place), the same general curve shape is observed (Fig. 3b, c and d), suggesting it reflects a general evolution in China. Prevalence of HBsAg in first-time donors was significantly higher than in the repeat-donors (Fig. 3b, *P* < 0.0001), in male donors than in female donors (Fig, 2c, *P* = 0.0001), and in native donors than in donors of other origins (Fig. 2d, *P* < 0.0001). The incidence of HBsAg (mean: 0.37 %) in repeat-donors decreased from 0.6 % in 2005 to 0.16 in 2013 and then rebounded to 0.38 % in 2014 (Fig. 3b).

HBsAg prevalence was examined in four age groups (18–25, 26–35, 36–45 and ≥46 years; Table 2). The overall HBsAg prevalence varied significantly according to age (*P* < 0001). Unexpectedly, the highest HBsAg prevalence (2.73 %) was found in the youngest age group (18–25 years), and progressively declined as age increased to the lowest level (1.71 %) in donors >45 years of age.

**Table 2:** HBsAg prevalence according to donor age groups

Age (years)	18–25		26–35		36–45		46–58		Total	
Donor type	HBsAg+/all	%	HBsAg+/all	%	HBsAg+/all	%	HBsAg+/all	%	HBsAg+/all	%
Male	3904/124,365	3.14	3601/156,658	2.30	1680/77,880	2.16	376/20,922	1.97	9561/379,825	2.52
Female	2015/92,680	2.17	940/56,170	1.67	543/31,383	1.73	137/9087	1.51	3635/189,320	1.92
First-time	5671/160,817	3.53	4218/121,447	3.47	2021/50,851	3.97	450/11,222	4.01	12,360/344,337	3.59
Repeat	248/56,228	0.44	323/91,381	0.35	323/91,381	0.35	63/18,787	0.34	836/224,808	0.38
Native	2567/73,214	3.51	1670/50,584	3.30	835/33,472	2.49	222/13,057	1.70	5294/170,327	3.11
Other origins	3352/143,831	2.33	2871/162,244	1.77	1388/75,791	1.83	291/16,952	1.72	7902/398,818	1.98
Overall	5919/217,045	2.73	4541/212,828	2.13	2223/109,263	2.03	513/30,009	1.71	13,196/569,145	2.32

### Prevalence of HBsAg between blood donors born before and after universal infant vaccination program implementation

In order to investigate whether the expected impact of the national neonatal vaccination program implemented in 1992 in blood donors was confirmed, the presumed vaccinated and non-vaccinated blood donor populations were separated according to being born before or after 1992. Blood donors born in 1992 or later who gave blood between 2010 and 2014 at 18–22 years of age were considered as vaccinated donors. Blood donors born before 1992 who gave blood between 2005 and 2013 were considered as non-vaccinated donors giving blood within the same age range. In first-time donor population aged 18–22 (Table 3), HBsAg prevalence was 3.61 %. There was a statistically significant difference in HBsAg prevalence between the populations born <1992 (3.51 %) and born ≥1992 (3.89 %, *P* = 0.005). In the population of first-time donors assumed vaccinated born ≥1992, HBsAg prevalence tended to increase with age from 3.15 to 4.72 % (*P* = 0.003–0.0001, 18 v.s. 21 or 22 years). There was no significant difference in HBsAg prevalence according to age in first-time donors born prior to 1992 (*P* = 0.228–0.74, 18 v.s. 20, 21 or 22 years). Between assumed vaccinated and non-vaccinated repeat-donor populations tested at the same ages (18–22, Table 4), the incidence of HBsAg seroconversion was significantly lower in donors born ≥1992 (0.27 %) than in those born prior to 1992 (0.57 %, *P* = 0.008). Within the population of repeat-donors, the incidence of HBV infection was found higher at age 22 either born prior to 1992 (0.55 %) or born ≥1992 (0.32 %) than that at age 18 born <1992 (0.2 %) or born ≥1992 (<0.01 %), respectively, but the difference was not statistically significant (*P* > 0.05). Combining first-time and repeat donors (Tables 3 and 4), the overall HBsAg prevalence calculated in younger donors aged 18–22 years (3.12 % [3689/11423]) was higher than in donors aged ≥23 (2.73 %) (Table 2, *P* < 0.001).

**Table 3:** Prevalence of HBsAg in first-time donors born before and after 1992 tested at the same ages

Age year	Born <1992		Born ≥ 1992		Overall		*P* value
	HBsAg+/total	%	HBsAg+/total	%	HBsAg+/total	%	inter-group
18	152/4600	3.30	162/5151	3.15	314/9751	3.22	0.656
19	373/10,158	3.67	280/8212	3.41	653/18,370	3.55	0.340
20	545/14,797	3.68	253/6029	4.20	798/20,826	3.83	0.080
21	612/17,982	3.40	191/3833	4.98	803/21,815	3.68	0.000
22	692/20,146	3.43	99/2097	4.72	791/22,243	3.56	0.002
All ages	2374/67,683	3.51	985/25,322	3.89	3359/93,005	3.61	0.005

Intra-population of first-time donors, *P* = 0.228–0.74 from 18 v.s. 20, 21 or 22 years born <1992; *P* = 0.003–0.0001, 18 v.s. 21, or 22 years born ≥1992

**Table 4:** Incidence of HBsAg seroconversion in repeat donors born before and after 1992 tested at the same ages

Age year	Born <1992		Born ≥ 1992		Overall		*P* value
	HBsAg+/total	%	HBsAg+/total	%	HBsAg+/total	%	Inter-group
18	1/490	0.20	0/318	<0.01	1/808	0.12	0.606
19	16/2042	0.78	5/1283	0.39	21/3325	0.63	0.163
20	26/4126	0.63	6/1620	0.37	32/5746	0.56	0.234
21	30/5868	0.51	1/1262	0.08	31/7130	0.43	0.034
22	43/7779	0.55	2/630	0.32	45/8409	0.54	0.334
All ages	116/20,305	0.57	14/5113	0.27	130/25,418	0.51	0.008

Intra-population of repeat donors, *P* = 0.202–0.298 from 18 v.s. 20, 21, or 22 years born <1992; *P* = 0.341–0.441from 18 v.s. 21 or 22 years born ≥1992

### Yield of HBV DNA positive but HBsAg negative blood donors

From January 2005 to December 2014, 475,538 samples with negative HBsAg and normal ALT (<50U/L) were further tested for HBV DNA by NAT. A total of 144 donations were identified as HBV DNA+/HBAg-, resulting in a yield of 1:3,302 seronegative blood donors (Table 5). According to follow-up testing results, 106 donors were identified as occult HBV infection (OBI), 11 as window-period infection (WPI) and 27 unclassified, respectively (Table 5). OBI and WPI frequencies were 1:4,486 and 1:43,231, but 27 HBV DNA+/HBAg- samples remained unclassified (1:17,613). OBI carriers were slightly, but not significantly, more frequent in male (0.020 %) than in female (0.015 %). Median age was 34 years and had low anti-HBs level (median 3.9 IU/ml). Viral load (median 23.1 IU/ml) of OBI samples was low ranging between undetectable (<10 IU/ml) and 2122 IU/ml. Of 76 HBV DNA+ samples available for genotyping, 58 samples (76.3 %) were identified as genotype B and 18 (23.7 %) as genotype C, respectively.

**Table 5:** Yield of HBsAg-/HBV DNA+ in blood donors^a^

Category	OBI	WPI	Unclassified	Overall
Samples	106	11	27	144
Yield	1/4486	1/43,231	1/17,613	1/3302
Gender (F/M)	29/77	5/6	7/20	41/103
Age range (median)	20–56 (34)	19–42 (28)	19–54 (28.5)	19–56 (32)
VL range (median, IU/ml)	0–2122 (23.1)	0–7321 (190.4)	0–2782 (36.4)	0–7321 (27.9)
Anti-HBc+/anti-HBs+/anti-HBe + (%)	2	0	0	2
Anti-HBc+/anti-HBs + (%)	34	0	0	34
Anti-HBc+/anti-HBe + (%)	4	3	0	7
Anti-HBc + only (%)	54	1	0	55
Anti-HBs + only (%)	8	0	0	8
HBeAg + only (%)	0	1	0	1
Anti-HBe + only (%)	0	0	0	0
No seromarker (%)	4 (primary OBI)	6	27	37

^a^HBV DNA was detected by the NAT from 475,538 blood donors with negative HBsAg and normal ALT level (<50U/L). HBV DNA+/HBsAg- carriers were detected 1–3 times for HBV DNA and sero-markers by the follow-up. The blood samples were detected for HBV DNA+/HBsAg- in follow-up samples were defined as OBIs, for seroconversion of HBsAg were defined as window period infections (WPIs), while no available of follow-up testing were defined as unclassified that might include false positive of HBV DNA samples

## Discussion

Shenzhen is a modern city close to Hong Kong, located in the Eastern part of Guangdong province, south China. Over 10 millions of people live in Shenzhen, 88.41 % of them being 15–59 years old. According to age distributions of blood donors, young adults with ages 18–35 years are the majority of the blood donor population, who contribute nearly 80 % of blood donations (Fig. 2). Shenzhen is also a younger city and approximately 70 % of blood donors are migrant citizens (Table 1) somewhat reflective of Chinese population as a whole [15].

In this study, half a million blood donors were screened for chronic, window period or occult HBV infections between 2005 and 2014 in Shenzhen, China (Tables 1 and 4, Figs. 1 and 3). An overall prevalence of HBsAg in blood donors was 2.9 % in 2005, declined to 1.8 % in 2010, then rebounded to 2.8 % in 2014 during past 10 years (Fig. 3). Observed HBV prevalence was significantly higher in first-time donors than in repeat-donors, in male than in female and in native than in donors of other origin, respectively (*P* < 0.0001). As expected, HBsAg prevalence in first-time donors significantly increased with age (Table 2). Low and stable prevalence of HBsAg in repeat donors reflected either new infections occurring between blood donation or higher sensitivity of assays over time.

The most surprising result in this study was the higher HBsAg prevalence in assumed vaccinated first-time donors (3.89 %) aged 18–22 between 2010 and 2014 than in presumed non-vaccinated donors in the same age range (3.51 %) tested at the same age between 2005 and 2009 (Table 3, *P* < 0.005). HBsAg prevalence in first-time donors born after 1992 tended to increase with age while in donors born earlier, no such difference in prevalence according to age was observed (Table 3). These apparently surprising results can be interpreted by examining the expected rate of vertical HBV transmission before 1992 and after 1992 when compliance was settling in and the changes in HBV infection in vaccinated Chinese over the age of 14. Between 1987 and 1992, the rate of HBV vaccination in China was estimated at 30 % [3, 16]. Assuming a prevalence of HBsAg of 15 % in the Guangdong province [16] and a 40 % rate of vertical transmission [17, 18] and 70 % of the population not vaccinated, the predicted incidence of HBsAg in children was 4.3 %, expected to rise somewhat when these children are tested between age 18 and 22. The observed HBsAg prevalence in such adults having become blood donors was 3.5 %, lower than anticipated. A possible explanation might be that among the 30 % vaccinated population, HBsAg positive women were selected for offspring vaccination.

Applying the same calculations to children born in 1992–1996 (blood donors of 18–22) during the ramp up of vaccination compliance from 30 to 70 %, the expected HBsAg rate is predicted to decline from 4.25 to 1.8 % (mean 3.0 %). This estimate is considerably lower than the 3.9 % observed (Table 3), suggesting that infections acquired by other means than MTCT played a role. In two large studies conducted in China and Taiwan, respectively, the prevalence of HBsAg between age 10 and 20–24 progressively increased in presumably or effectively vaccinated general populations from less than 1 % to approximately 3 % [3, 9]. In the Taiwanese study, evidence of contact with HBV after age 14 when the prevalence of anti-HBs has declined below 20 % is provided by increasing prevalence of HBsAg from 1 to 3 %, anti-HBc from 2 to 11 % and anti-HBs from 19 to 68 % by age 24 [9]. This data strongly suggests that protection to HBV genotype B infection provided by genotype A1 vaccine declines with age and translates into seroconversion to HBsAg and/or anti-HBc or natural anti-HBs boost when in contact with HBV in a high prevalence of chronic infection area [12, 19]. The HBsAg prevalence observed in our study is quite compatible with this observation. This different distribution might be related to the progressive decline of anti-HBs vaccine-related protection leaving these vaccinated donors at higher risk of infection as they grow older and as previously reported [12]. Other factors may also interfere such as the type of vaccine utilized (plasma derived or recombinant) but here are probably insignificant. However, between age 18 and 22, repeat donors presumably vaccinated had 0.3 % incidence of HBV infection, nearly half the rate observed in older donors, presumably not vaccinated (Table 4). This incidence of HBsAg seroconversion did not significantly increase with age (Tables 3 and 4) [12]. This observation is compatible with frequently abortive infection when vaccinees with low level of anti-HBs are exposed sexually to high levels of HBV [19]. The contrast in donors born since 1992 between the high prevalence of HBsAg and relatively few recent infections suggests chronic infections being acquired early in life presumably out of non compliance to vaccination or failure of the immunization and relatively effective protection against new infection likely related to sexual activity. In the cohort of donors born before 1992, it is possible that a fairly high proportion of them had been vaccinated ahead of the generalization of the intervention explaining the relatively low prevalence of chronic HBV infection.

In recent years, occult hepatitis B virus infection (OBI) attracted considerable attention with regard to blood safety [5, 20], and was found the major remaining residual risk of transfusion-transmitted viruses [14, 15]. In Shenzhen blood donor population, the yield of 1:3,302 was detected for HBV DNA+/HBsAg-, included relatively frequent OBIs (1:3,835) and few cases of window period infection (1:67,934), although these frequencies might be modified if all yield samples had been categorized (Table 4). The molecular biological features of OBIs in blood donors have been well characterized in East Asia [12–14, 21]. This transfusion risk could be minimized but not completely eliminated by NAT screening in blood donation due to insufficient sensitivity of current assays [6, 7].

Since hepatitis B vaccination became mandatory for all newborns within 24 h of birth nationwide in 1992, this universal vaccination program decreased the HBV prevalence to 1 % in vaccinated children aged <5 years and led to a reduction of HBV prevalence to 7.2 % in the general population of China [1]. HBV prevalence reported from Shenzhen blood donors including vaccinated first-time donors is clearly lower than that from the general Chinese population, but still remains much higher than expected in vaccinated children. The potential causes of HBV infection in blood donors, particularly in vaccinated population may originate from the following options [22]. (1) Low level or undetectable anti-HBs. Previous study confirmed that there is among adequately vaccinated newborn a small portion (3-7 %) of non- or low-responders [23]. In addition over 50 % vaccinated children no longer carry detectable anti-HBs when reaching 11–17 years [9, 24]. In our previous study of 1494 vaccinated blood donors aged 18–21 years in Shenzhen, Southern China, we found approximately 29 % of donors with no detectable HBV markers and 40 % of them carrying anti-HBs levels <100 IU/L [12]. Those vaccinees with low level of anti-HBs are susceptible to infection associated with breakthrough or leading to occult HBV infections as described previously [12, 25, 26]. (2) HBV genotype A2 vaccine efficacy. Current hepatitis B recombinant S protein vaccines are of genotype A2. A study found that 6 of 9 vaccinated blood donors were identified as OBIs [19]. Five of these donors had anti-HBs <100 IU/L and had been infected with non-A2 (genotype B, C, F or D) or mixed HBV strains, which suggested that protection offered by genotype A2 hepatitis B vaccine might not be fully effective for individuals exposed to non-A2 strains such as genotype B or C prevalent in China by close contact with sexual partners carrying high HBV load. (3) A previous study conducted in vaccinated blood donors revealed a prevalence of anti-HBc increasing with age consistent with an increasing cumulative HBV exposure [12], suggesting that those with low level immune response were insufficiently protected when in contact with high HBV DNA load mainly through sexual activity [19]. Many studies showed the decline and the high percentage of undetectable of anti-HBs in vaccinated people over 14 years of age, including blood donors [9, 12, 27]. A vaccine boost in adolescents has been considered and its efficacy was demonstrated [28–30]. However the justification of such strategy remains controversial and its implementation still under consideration.

## Conclusions

In this study, we observed that the prevalence of HBsAg was below 2 % in 2010 and rebounded to nearly 3 % in 2014, which might be attributed to a higher incidence of HBV infection but also to a change of either test performance or record keeping of donor exclusion with HBsAg rapid tests. The modest differences in HBsAg prevalence prior to or since 1992 can be explained by the slow and progressive increase in vaccination compliance in the Guangdong province. The same study conducted in blood donors born after 2002 when compliance reached over 90 % might better reflect the impact of HBV vaccination on the safety of the blood supply. From a transfusion safety point of view, an HBV vaccine boost injected between age 15 and 17 years (prior to starting sexual activity) might be considered to limit the potential for sexually related new infections. A powerful clinical trial comparing the incidence of HBsAg and anti-HBc in young adults having received or not a vaccine boost around age 14 years would be highly informative.

## Abbreviations

ALT: Alanine aminotransferase
anti-HBc: Antibody to hepatitis B virus core antigen
anti-HBe: Antibody to hepatitis B virus e antigen
anti-HBs: Antibody to Hepatitis B virus surface antigen
EIA: Enzyme immunoassay
HBeAg: Hepatitis B virus e antigen
HBsAg: HBV surface antigen
HBV: Hepatitis B virus
HCV: Hepatitis C virus
HIV-1: Human immunodeficiency virus-1
NAT: Nucleic acid testing
OBI: Occult HBV infection
PCR: Polymerase chain reaction
WPI: Window period infection
